# Discovery and study of cutaneous leishmaniasis in Karamay of Xinjiang, West China

**DOI:** 10.1186/2049-9957-2-20

**Published:** 2013-09-08

**Authors:** Li-Ren Guan, Yuan-Qing Yang, Jing-Qi Qu, Hao-Yuan Ren, Jun-Jie Chai

**Affiliations:** 1 National Institute of Parasitic Diseases, Chinese Center for Disease Control and Prevention, Shanghai 200025, PR China; 2 General Hospital of Xinjiang Petroleum Bureau, Karamay 834000, PR China; 3 Center for Disease Control and Prevention of Xinjiang Uygur Autonomous Region, Urumqi 830002, PR China

## Abstract

Cutaneous leishmaniasis (CL) was discovered in the farms of the Karamay suburb, Xinjiang Uygur Autonomous Region in the 1990s. Between 1992 and 1994, a house-to-house survey revealed a prevalence of 1.0-1.6% in the residents. The clinical types of skin lesions included papule, plaque, ulcer and nodular prurigo. Observations verified that, in some cases, the skin lesions healed spontaneously in 10–14 months, whilst in other cases, they persisted for several years. Sporadic cases of CL have continued to appear at the dermatology clinic of the local hospital since 2000. *Phlebotomus wui* (*Ph. wui*), subgenus *Larroussius* was confirmed as the transmitting vector. The causative agent is *Leishmania infantum sensu lato*.

## Multilingual abstracts

Please see Additional file 1 for translations of the abstract into six official working languages of the United Nations.

## Review

Visceral leishmaniasis was one of the most important parasitic diseases, rampant in the plain region of eastern China in the early 20^th^ century. Although it was virtually under control in that region through active detection of human infections for treatment and vector control since the 1960s, the disease has continued to occur sporadically in the western mountainous and desert areas. Considering that cutaneous leishmaniasis (CL) has been endemic in the neighbouring countries such as Afghanistan, Kazakhstan and other central Asian Republics of the former USSR, whether CL exists in western China is of interest. Chtcherbakoff (1930) reported two cases of oriental sore in Kashgar, Xinjiang. There have been no further reports of CL since then until the 1980s [1]. Zhang (1983) reported a case of dermal leishmaniasis, which was inferred to be caused by *Leishmania donovani* in Shawan, Xinjiang [2]. Ren (1984) clinically diagnosed eight cases of oriental-sore-like CL [3], but *Leishmania* amastigote was not found in the smears of the skin lesions. In the autumn of 1988, we made definitive diagnosis microscopically for two cases of CL [4], and also found *L. turanica* in the subcutaneous ear tissue of the great gerbil at the Xiaoguai farm in Karamay, Xinjiang. Inoculation of a human volunteer with the *Leishmania* isolate was found to cause skin lesions [5]. It is therefore necessary to determine whether *Leishmania turanica* (*L. turanica*) is the pathogen of the local human CL and to determine its clinico epidemiology, i.e., prevalence, pathology, diagnosis, clinical manifestations, therapy and vector. This article summarises and discusses the major findings relating to CL since the 1980s.

## Survey of epidemiology

A three-year survey indicated that the annual incidence of CL in Karamay was 1.6% (36/2260), 1.0% (14/1416) and 1.6% (24/1510) for the successive years from 1992 to 1994, respectively. The yearly difference was related to the resident versus migrant population ratios.

Based on the 1992 survey, the infection rate was as high as 13.1% (8/61) among those who migrated from non-endemic areas and resided in the area for less than two years, while it was only 1.3% (28/2199) for the indigenous residents [6]. Of the 22 CL cases diagnosed in April 1993, 16 of those were migrants who came to the area in the last two years. The survey also showed no family aggregation of CL. According to the Dermatology Department of the Karamay Workers Hospital’s records available since 2000, there have been individual visits to seek medical advice about CL (Li Fan, personal communication, 2013).

## Clinical manifestations and pathology

The analysis of clinical manifestations for 90 cases of CL patients indicated that four types of skin lesions, viz., papule, plaque, ulcer and nodular prurigo existed, of which papule and ulcer were the more common. Generally, one to five skin lesions were visible. A few cases had abscess and pustule-like lesions. None of the 90 CL cases had clinical manifestations or any history of visceral leishmaniasis (VL). Twenty-six patients were subjected to blood examinations. Leukocyte count and differential count were within the normal range. Fifteen cases were examined by B-mode ultrasonography, showing no significant abnormality in the size and thickness of the spleen [6].

**Figure 1:**
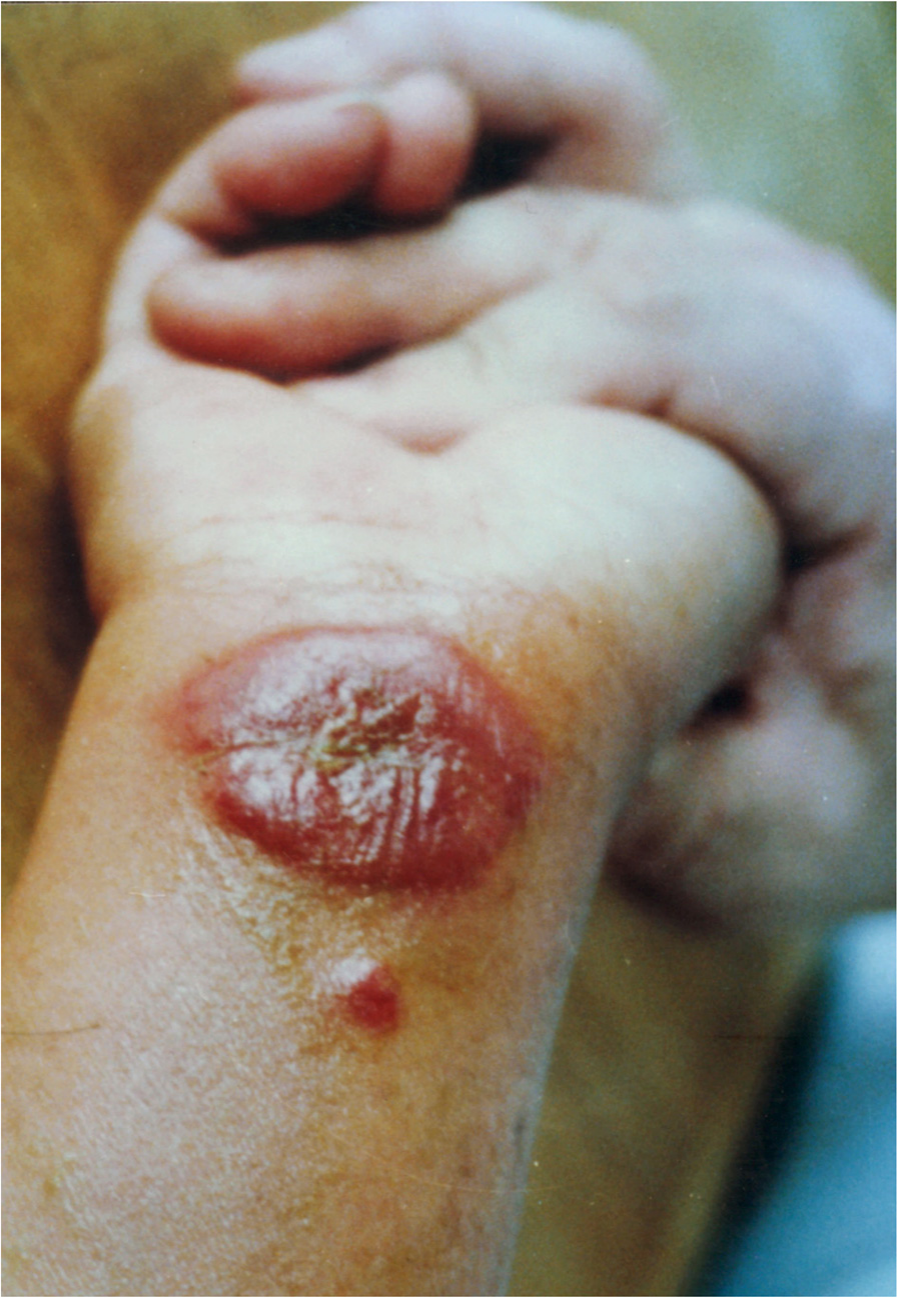
**A large ulcer on the forearm with a small nodule both containing *Leishmania *amastigotes.**

**Figure 2:**
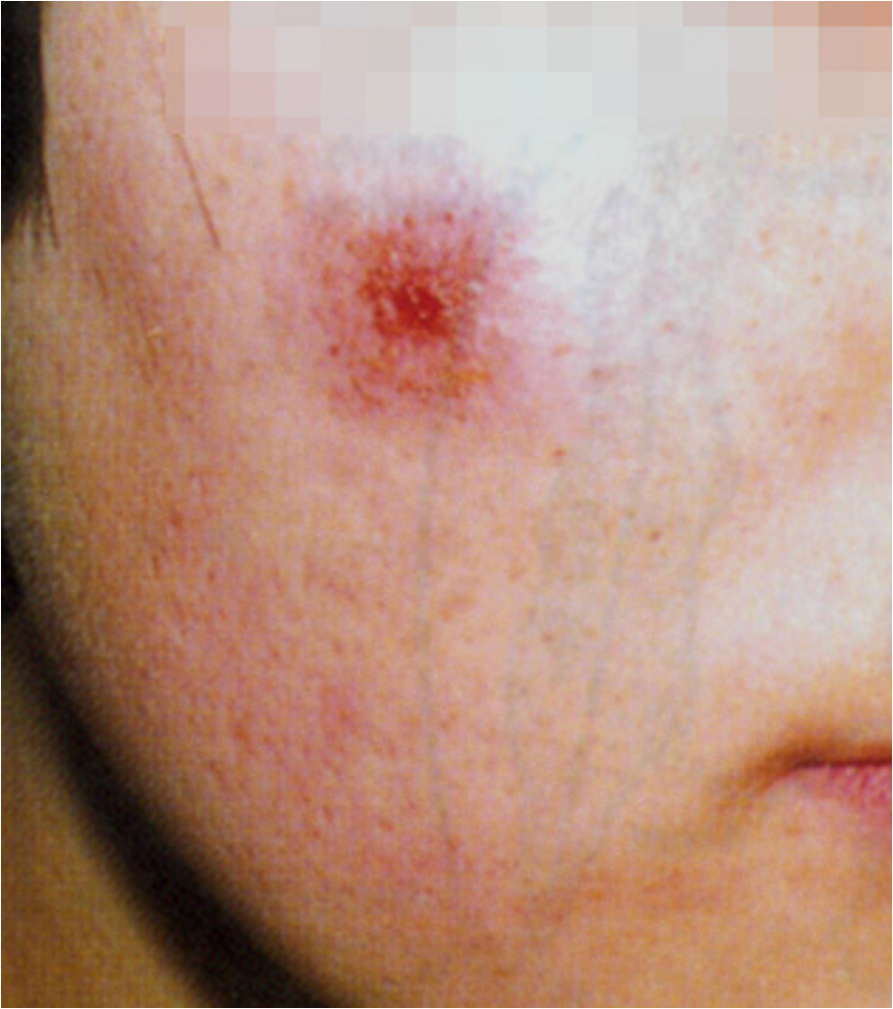
An ulcer on the face.

**Figure 3:**
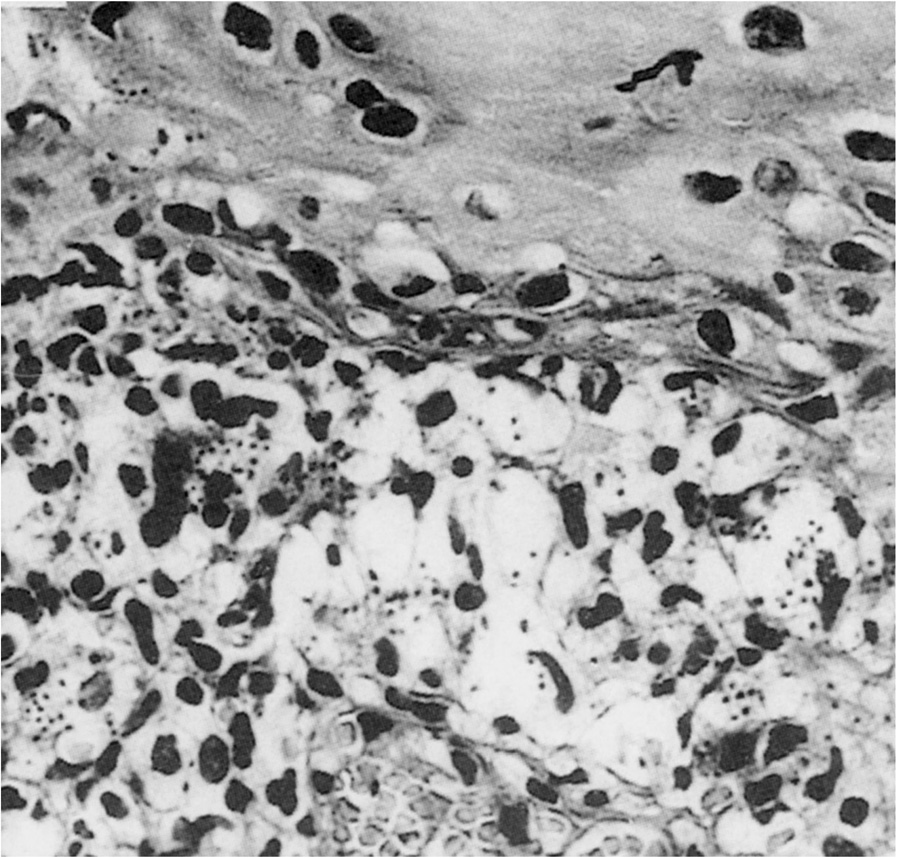
**Histopathology of a section from the facial ulcer (Figure**2**) showing numerous *Leishmania *parasites in dermis (HE staining, x 400)**

Cutaneous lesions showed gross and histopathological changes. *Papule and plaque*: presence of numerous parasites in infected macrophages and infiltration of inflammatory cells. Concurrently, collagenous fibres decreased or disappeared. *Ulcer*: necrosis and detachment occurred in some ulcers, and *Leishmania* and debris of inflammatory cells were found in the necrotic materials. Granuloma was formed on the edge of ulcers. Congestion, edema and infiltration of inflammatory cells appeared in the dermal tissue and *Leishmania* amastigotes were detected in the inflammatory foci (see Figures 1, 2 and 3). *Nodular prurigo:* TB tubercle-like nodules composed of epithelioid cells, fibroblasts, giant cells and lymphocytes were observed in the dermal tissues. Newly-formed blood vessels and proliferation of collagenous fibres were seen in the nodules. *Leishmania* amastigotes were rarely detected [7].

A follow-up study of three patients showed that the skin lesions disappeared spontaneously in 10–14 months without specific treatment. In some other patients, skin lesions had already been in existence for several years before they sought medical attention. *Leishmania* were observed in the skin tissues.

## Diagnosis

The 50 CL cases diagnosed initially by histopathological examination of the skin lesions were re-examined by the skin smear technique. Typical *Leishmania* amastigotes were seen in 11 out of the 31 cases with skin papule or ulcer (35.5%), but not in any of the other 19 cases with plaque or nodular prurigo. Immunodiagnosis revealed a seropositive rate of 83.3% (25/30) by sandwich Dot-ELISA to detect circulating antigen/antibody-antigen complex [8].

### Treatment

Ten cases (two with papule, seven with ulcer and one with nodular prurigo) with 67 skin lesions were selected for liquid nitrogen cryotherapy. After two to three days, crust was formed. Two months later, decrustation occurred and the lesion was healed. Follow-up examinations of five cases one to two years after treatment showed that the gross appearance of the former lesion sites were normal. However, a few *Leishmania* were found in the continuous histological sections, suggesting that although cryotherapy can heal the lesions rapidly or block the development of the dermal disease, it cannot eliminate *Leishmania* completely from the skin lesions [9].

## Parasite biology

### Growth and propagation of *Leishmania* in Novy-McNeal-Nicolle (NNN) medium

The homogenates of skin lesions from 21 cases were individually inoculated into Novy-McNeal-Nicolle (NNN) medium. Few promastigotes were observed only in three cases; promastigotes from two cases disappeared after passages. Only one isolate (KXG-Liu) survived in the medium. The difficulty to isolate *Leishmania* from these skin lesions of CL cases is in sharp contrast to the ease of growing *L. turanica* from ear skin tissues of great gerbils in the same medium [5,10], suggesting that it is not the causative parasite of the 21 human CL cases.

### Infectivity to experimental animals and pathological changes

Two samples (KXG-Xu and KXG-Zhang) of CL skin lesion homogenates and KXG-Liu promastigotes were inoculated separately into the abdominal cavity or testes of *Lagurus lagurus* and *Cricetulus brabensis*. Autopsy was conducted after 96–302 days post-infection. The gross examination showed visceral infection in both species of the experimental animals, just like inoculation of these animals with *L. donovani* (LD) and *L. infantum* (LI). A histopathological study showed that *Leishmania* parasites were present in Kupffer cells, reticuloendothelial cells in bone marrow, spleen, macrophages and dendritic cells in lymph sinus of medulla and cortex of lymph nodes, and glomeruli, epithelial cells of proximal convoluted tubules and mesenchyme of kidney. Ultrastructural studies of the plasma cells showed that their rough endoplasmic reticulum expanded markedly, suggestive of activation of the immune cells [10]. Promastigotes emerged and grew after inoculation of infected tissues into NNN medium. BALB/c mice were not susceptible when inoculated subcutaneously or intraperitoneally with *Leishmania* amastigotes from the patients’ skin lesions [10].

Thus, the *Leishmania* from the skin lesions of the CL patients produced systemic visceral infection only in the susceptible animals used, which were different from the skin lesions produced by *Leishmania tropica* (*L. tropica*) or *L. turanica* in BALB/c mice [5,11].

When two *Macaca rhesus* monkeys were inoculated subcutaneously with KXG-Liu and KXG-Xu, macule and ulcer developed at the sites of inoculation. The skin lesions disappeared approximately three months later. However, numerous *Leishmania* parasites were observed in the macrophages in the muscular layer of the subcutaneous blood vessel wall tissue, but none in the visceral tissues when examined by histological sections 12–14 months after infection. Visceral tissues were also culture-negative, further indicating that the *Leishmania* from Karamay are cutanotropic in primate hosts [12].

### Morphology of *Leishmania*

In the longitudinal section, the size of amastigote of KXG-Liu isolate was (3.68±0.6) μm × (1.80±0.28) μm and the size index was 6.16±1.56 by transmission electron-microscopy, similar to the observation under light microscope. The average number of submembrane microtubule of *Leishmania* in transverse section was 74±7 [10].

### Genotype DNA analysis on *Leishmania*

The genotype analysis for nDNA and kDNA of KXG-Liu isolate was conducted in 1994 [13]. The results indicated a high homology of DNAs of the isolate to the viscerotropic LI elsewhere in Xinjiang (MHOM/CN/80/801, MHOM/CN/90/901). Subsequently, Southern blot analysis of nDNAs from KXG-Liu and KXG-Xu for gp63 gene RFLP showed high homology to the Chinese LI (MHOM/CN/86/SC) and the World Health Organization (WHO) LI reference strain (MHOM/TN/81/LEM235), differing from *L. tropica* and *L. turanica*[14]. Phylogenetic analyses of gene sequences from these Karamay CL isolates distinguish them from three variants of viscerotropic *L infantum* from China and elsewhere, but segregate all together into the same clade of *L donovani/infantum* complex [15]. Pulse field gel electrophoresis was used to analyse the karyotypes of the two isolates, showing its similarity to the LI from the bone marrow of a VL patient in Shanshan County, Xinjiang (MHOM/CN/90/901) [16]. The two Karamay CL isolates were typed by zymodeme analysis to belong to *L. donovani* (MON-138) by professor Jean-Pierre Dedet, Ecological Medicine Lab, Montpellier University (unpublished, 1997).

## Studies on vector sandfly

### Sandfly species in CL endemic and non-endemic areas

Sandflies were collected by a suction tube, by a funnel trap or by sticky paper during the months of June to August in 1987–1993. Identification of species was based on morphological features. The common sandfly species found in Karamay were *Phlebotomus mongolensis* (*Ph. mongolensis*) and *Ph. wui*, accounting for 45.9% (4105/13733) and 24.2% (3318/13733), respectively. The rest 29.9% (4105/13733) were a mixture of *Ph. andrejevi, Ph. caucasicus, Sergentomyia minutus sinkiangenesis* and *S. arpaklensis*. The survey in the adjacent four counties, i.e., Wushu, Fukang, Qitai and Yiwu, 100–400 km away from Karamay, showed different sandfly species. In Wushu, Fukang and Qitai, *Ph. mongolensis* and *Ph. andrejevi* were the dominant species, accounting for 90.1% to 97.9%, respectively, while *Ph. wui* constituted only 0–2.1%. The dominant species in Yiwu was *Ph. alexandri*, accounting for 70.8% [17,18].

### Relationship between sandflies and humans

*Phlebotomus mongolensis* and *Ph. wui* in Karamay were anthropophilic, accounting for 43.1% (1111/2580) and 30.3% (773/2580), respectively, while *Ph. andrejevi* only accounted for 0.8% (21/2580) of the total catches indoors. A total of 2767 man-biting sandflies were captured by human-bait in the field, of which the proportion of *Ph. wui* was 55.7% and of *Ph. mongolensis* was 39.0%; while the two species captured indoors accounted for 61.4% and 35.7%, respectively [18].

### Natural infection of sandflies and identification of parasite species

The natural infection rate of *Ph. wui* with promastigotes was 5.9% (58/985) for those captured in the field and 2.9% (13/449) for those in the residential sites. The gut of positive flies was full of promastigotes when examined microscopically. Parasites were found in the pharynx, mouth and proboscis of 34 sandflies (47.9%) [18]. Inoculation of the promastigotes from 36 infected sandflies into NNN medium resulted in no viable cells, except one (KXG-65) [19], exactly as observed with the amastigotes from CL lesions in the same medium [10]. In a separate study, starved *Ph. wui* females were found to acquire infection when allowed to feed on *L. turanica*-infected BALB/c mice, as indicated by microscopy of fed flies after dissection and the successful cultivation of promastigotes from 10 such flies [19]. The results from the above experiments suggest that the parasite species in naturally infected *Ph. wui* is not *L. turanica*, which is found in the local great gerbils.

Inoculation of promastigotes from naturally infected *Ph. wui* directly into the footpad of 14 BALB/c mice produced no skin infections, although few *Leishmania* amastigotes were found in the spleen tissue smears. *Lagurus lagurus* were then inoculated intraperitoneally with the same fly-derived promastigotes – of the five survived for more than three months, four developed visceral infection showing pathological changes as those caused by LI. When small pieces of spleen from the infected animal were inoculated into NNN medium, two isolates (KXG-918, KXG-927) were obtained for continuous passages. When these two isolates and KXG-65 were inoculated intraperitoneally to *C. brabensis*, the infection site and pathology were identical to those isolated from the local human CL caused by LI [20]. Subcutaneous inoculation of a monkey with sandfly-derived promastigotes also exclusively caused skin lesions [12].

Hybridisation by using 32p labelled gp63 gene as probe suggested that three isolates (KXG-918, KXG-927 and KXG-65) of promastigotes from naturally infected *Ph. wui* were highly homologous to LI of Xinjiang, but showed marked variation in nDNA hybridisation map as compared with those of both *L. turanica* and *L. tropica*[14,20]. The zymodeme of the three isolates, MON-138, was identical (Dedet, unpublished, 1997).

*Ph. mongolensis* and *Ph. andrejevi* prevalent in Karamay are the vectors for transmission of *L turanica* in great gerbils, as shown by examining 16 independent isolates of this species [5,19].

## Conclusion

Based on the information presented, CL is expected to remain endemic in Karamay, Xinjiang. It is caused by a variant of *L. infantum*, which is transmitted by *Ph. wui*. Different variants of LI are responsible for VL and CL in Xinjiang [15]. The current discovery is consistent with previous findings that LI, normally the causative agent of VL, also produces CL in 11 countries of the Eastern hemisphere [21-23]. The WHO publication on the control of leishmaniases places *L. infantum* into the *L. donovani* complex [22]. Therefore, the isoenzyme electrophoresis results noted by Dedet based on the human and sandfly isolates that we provided showed no contradiction with our results of genotype DNA analysis. In three countries in the Western hemisphere, *Leishmania chagasi* (*L. chagasi*) was thought to cause CL [21]. Rioux and associates examined the isoenzyme gene loci for 137 isolates from six countries in Latin America and proved that those isolates and LI had the same zymodemes [24,25]. LI in the Mediterranean basin was reported to have two different pathogenic strains in humans with and without immunodeficiency syndromes. Strain with zymodeme MON-1 (LON-49) mainly causes VL, while those of zymodemes MON-24, -29, -33, -78, -II, -III are associated with CL [26]. Angelica *et al.* indicated that differences exist in genotypes between the isolates of viscerotropic and cutanotropic LI [27]. Based on sequence variations, Waki *et al.* designated CL LI (KXG-Lu, -Xu and -Liu) and sandfly isolate (KXG-65) in Karamay as *L infantum* var. 7 and differentiated it from other human LI variants [15]. Further studies are needed to elucidate the distribution and phylogenetic relationships of different LI variants in China.

The LI causing CL in Karamay was identical with its counterpart elsewhere in the following characteristics: 1. Cultivation of LI causing CL directly from patient and sandfly samples *in vitro* is difficult, but made possible after inoculation of susceptible animals for visceral infection [28,29]; 2. The LI from Karamay produced visceral infection, which is rather marginal in inbred BALB/c mice, but is significant in susceptible animals, such as Chinese hamsters [30,31]; and 3. The vector for the LI in Karamay is *Ph. wui*, whereas it is *Ph. perniciosus* in Malta and Spain, *Ph. pefiliewi* in Italy and Algeria, *Ph. ariasi* in France and Spain, *Ph. neglectus* in Greece and *Ph. Tobbi* (Turkey) in the Mediterranean basin, though all in subgenus *Larroussius*[20,25,28,30-33].

Although the great gerbils are widely distributed in Xinjiang north of the Tian Shan Mountain, *L. major* has not been found in rodents or humans [5,34], and there is no record of CL in the south of the Tian Shan Mountain. Therefore, there is no clear evidence for importation of this species to account for CL endemicity in Xinjiang.

While *L. turanica* in great gerbils in Karamay has the capacity to cause human infection experimentally [5], the parasite species has not been isolated from the skin of CL patients. This may be related to the activity of its natural vectors: *Ph. andrejevi* is active mainly near the rodent burrow in the field and it is difficult to find infected *Ph. mongolensis* in residential sites [18,19]. Thus, *L. turanica* does not contribute to the endemicity of local CL. Circumstantial evidence suggests that CL in Karamay is probably a zoonotic disease based on the following considerations: 1. The area was previously a barren desert and CL patients began to emerge after human settlement for economic activities; 2. CL occurs only sporadically and shows no evidence of family aggregation; and 3. *Ph. wui* is an exophilic species, showing high natural infection rate of LI among those in the uninhabited desert area. It is thus more likely that the source of parasites for sandfly infections is a wild animal reservoir instead of CL patients. Apparently, further investigation is needed to elucidate its prevalent scope and reservoir hosts.

## Competing interests

The authors wish to declare that they have no competing interests.

## Authors’ contributions

LRG and YQY designed the study and wrote the paper. LRG, JQQ and YQY collected and analyzed the data. HYR and JJC analyzed the clinical data. All authors read and approved the final manuscript.

## Supplementary Material

Additional file 1 Multilingual abstracts in the six official working languages of the United Nations. Click here for file
