# Supplementary material for: Discovery and study of cutaneous leishmaniasis in Karamay of Xinjiang, West China
Source: Infect Dis Poverty. 2013 Sep 8;2:20. doi: 10.1186/2049-9957-2-20 (PMC3856449; doi:10.1186/2049-9957-2-20)
Supplement: Additional file 1 — Multilingual abstracts in the six official working languages of the United Nations. [file 2049-9957-2-20-S1.pdf]

Translation of the abstract into the six official working languages of the United Nations

اكتشاف ودراسة داء الليشماتيات الجلدي في مدينة كاراماي في شينجيانج، في غرب الصين

لي – رين جوان، يوان – كينج يانج، يانج- كي كو، هاو- يوان رين و جون- جي تشاو

ملخص

تم اكتشاف داء الليشماتيات الجلدي (CL) في المزارع الواقعة في ضاحية كاماراي في إقليم شينجيانج يوجوار المتمتع بالحكم الذاتي في تسعينات القرن العشرين. فيما بين عامي 1992 و1994، كشفت دراسة تمت من بيت إلى آخر عن تفشي بنسبة 1.0 – 1.6% بين السكان. وقد تضمنت الأنواع العيادية من تضرر الجلد، وجود بثرات وصفحات وحُكاك قرْحي وعُقَيْدي. وقد أكدت الملاحظات أنه، في بعض الحالات، تم شفاء تضرر الجلد بصورة تلقائية من 10 – 14 شهر، بينما في حالات أخرى، استمرت لعدة سنوات. واستمرت الحالات المتقطعة لداء الليشماتيات الجلدي في الظهور في عيادة الأمراض الجلدية في المستشفى المحلي منذ عام 2000. وقد ثُبُتِ أن الفاصدة (جنس الذباب الفاصد) (Ph. Wui)، والجُنَيْس لاروسيوس (*Larroussius*) هي الحشرة الناقلة للمرض. والعامل المسبب هو *Leishmania infantum* *sensu lato*.

Translated from English version into Arabic by Reham Hussien, through

TRANSLATORS WITHOUT BORDERS

中国新疆克拉玛依皮肤利什曼病的发现和研究

Li-Ren Guan, Yuan-Qing Yang, Jing-Qi Qu, Hao-Yuan Ren and Jun-Jie Chai

摘要

1990 年代在新疆维吾尔自治区的克拉玛依郊区发现有皮肤利什曼病的流行。1992-1994 年经挨户调查，该病的流行率为 1.0%-1.6%。皮肤损害有丘疹、脓肿、溃疡和结节性痒疹型等 4 种不同的临床类型。经观察，有些患者的皮损可在 10-14 个月自愈，但有些患者的皮损可持续数年。自 2000 年以来，当地仍有散在病例发生。皮肤利什曼病的病原体是婴儿利什曼原虫，媒介为劳蛉亚属的吴氏白蛉。

Translated from English version into Chinese by Guan Li-ren, through

Découverte et étude de la leishmaniose cutanée à Karamay dans le Xinjiang (Ouest de la Chine)

Li-Ren Guan, Yuan-Qing Yang, Jing-Qi Qu, Hao-Yuan Ren et Jun-Jie Chai

Résumé

Des cas de leishmaniose cutanée ont été découverts dans les années 1990 dans des fermes des faubourgs de Karamay, dans la Région autonome ouïgoure du Xinjiang. Entre 1992 et 1994, une enquête en porte à porte a révélé une prévalence de 1,0 à 1,6 % de la maladie parmi les habitants de ces localités. Les types cliniques des lésions cutanées étaient des papules, des plaques, des ulcérations et un prurigo nodulaire. Des observations ont permis de constater, dans certains cas, une résolution spontanée des lésions cutanées en 10 à 14 mois, alors qu'elles persistaient pendant plusieurs années dans d'autres cas. Des cas sporadiques de leishmaniose cutanée se présentent encore à la clinique de dermatologie de l'hôpital local depuis 2000. Le vecteur de transmission identifié est *Phlebotomus wui* (*Ph. wui*), sous-genre *larroussius*. L'agent pathogène est *Leishmania infantum sensu lato*.

Translated from English version into French by Suzanne Assenat, through

TRANSLATORS
WITHOUT BORDERS

Обнаружение и изучение кожного лейшманиоза в г. Карамай, Синьцзян-Уйгурский автономный район Западного Китая

Ли-Рен Гуан, Юан-Кинг Янг, Джинг-Ки Ку, Хао-Юан Рен и Джун-Джи Чай

Краткий обзор

Кожный лейшманиоз (КЛ) был впервые обнаружен в 90-х годах прошлого столетия в сельских хозяйствах пригорода г. Карамай Синьцзян-Уйгурского автономного района. Опрос населения, проведенный в период с 1992 по 1994 год, выявил распространение этого заболевания среди 1,0-1,6% жителей города. Наблюдаемые клинические типы поражения кожи: бугорки, сыпь, незаживающие раны и узловатое пруриго. В период наблюдения было отмечено, что в некоторых случаях заживление пораженных участков наступает самопроизвольно спустя 10-14 месяцев, в то время как в других случаях кожные поражения не проходили в течение нескольких лет. Спорадические случаи кожного лейшманиоза появлялись в клинической практике дерматологического центра местной больницы с 2000 года. В качестве переносчика заболевания был определен *Phlebotomus wui* (*Ph. wui*) подрод *Larroussius*. При этом, возбудителем заболевания является *Leishmania infantum sensu lato*.

Translated from English version into Russian by Tatyana Kovaleva Modesto, through

TRANSLATORS WITHOUT BORDERS

Descubrimiento y estudio de leishmaniasis cutánea en Karamay, Xinjiang, Oeste de China

Li-Ren Guan, Yuan-Qing Yang, Jing-Qi Qu, Hao-Yuan Ren y Jun-Jie Chai

Resumen

La leishmaniasis cutánea (LC) se descubrió en las granjas del suburbio de Karamay, en la Región Autónoma de Xinjiang Uygur en la década de 1990. Entre 1992 y 1994, una encuesta realizada de puerta en puerta mostró una prevalencia de 1,0-1,6% entre los residentes. Los tipos clínicos de lesiones en la piel incluían pápulas, placas, úlceras y prurigo nodularis. A través de las observaciones se confirmó que, en algunos casos, las lesiones en la piel se curaron de forma espontánea luego de 10-14 meses, mientras que en otros casos, persistieron durante varios años. Han seguido apareciendo casos esporádicos de LC en la clínica dermatológica del hospital local desde el año 2000. Se confirmó al *Phlebotomus wui* (*Ph. wui*), subgénero *Larroussius*, como el vector de transmisión. El patógeno es *Leishmania infantum sensu lato*.

Translated from English version into Spanish by Maria Arias, through

TRANSLATORS WITHOUT BORDERS
